# Perceived clinician stigma and its impact on eating disorder treatment experiences: a systematic review of the lived experience literature

**DOI:** 10.1186/s40337-024-01128-3

**Published:** 2024-10-16

**Authors:** Gabriel Lubieniecki, Anne Nileshni Fernando, Alisha Randhawa, Sean Cowlishaw, Gemma Sharp

**Affiliations:** 1 Department of Neuroscience, School of Translational Medicine, Monash University, 99 Commercial Road, Melbourne, VIC 3004 Australia; 2 Turner Institute for Brain and Mental Health, Monash School of Psychological Sciences, Melbourne, Australia

**Keywords:** Eating disorders, Clinician stigma, Patient experience, Therapeutic alliance, Treatment barriers

## Abstract

**Objective:**

This systematic review examines the literature regarding perceived clinician stigma and treatment experiences of adult patients with eating disorders, emphasising lived experience perspectives.

**Method:**

A systematic search was conducted across MEDLINE, EMBASE, PsycINFO, and the Cochrane Central Register of Controlled Trials [CENTRAL] to identify studies published from 1 January 2000 until 24 March 2024 that explored patient experiences of clinician attitudes and behaviours in eating disorder treatment. Eligible studies included those reporting on perceived clinician stigma and impacts on treatment outcomes.

**Results:**

There were 11 studies that met the eligibility criteria. The studies encompassed various diagnoses, locations, and healthcare settings, reflecting a broad spectrum of experiences and contexts within treatment of eating disorders. Four key themes emerged across the studies: treatment engagement, where perceived clinician stigma led to reduced patient involvement; therapeutic alliances, with stigma compromising relationships between patients and healthcare providers; barriers to treatment and care, where stigma heightened obstacles to accessing support; and weight stigma.

**Discussion:**

Despite the overall scarcity of evidence, these qualitative studies provide evidence of impacts of perceived clinician stigma on patient experiences in eating disorder treatment. These findings provide an initial understanding of negative effects of clinician attitudes such as dismissiveness and invalidation, which may hinder treatment adherence and therapeutic outcomes. Beyond addressing stigma, future research should explore how clinician behaviours can foster positive treatment experiences, such as patients feeling heard, respected, and understood. Clinicians’ reflective practices should focus on improving therapeutic alliances and fostering more inclusive, patient-centred care. Future studies should prioritise mixed-methods approaches to investigate how clinician stigma and positive care experiences influence treatment engagement, recovery trajectories, and long-term outcomes.

## Abstract (plain language summary)

This review examines how adults with eating disorders perceive their treatment from doctors and other healthcare providers, focusing on feelings of stigma or negative judgment. We identified 11 relevant studies that discuss patient experiences with clinician attitudes in eating disorder treatment, covering various eating disorder diagnoses and treatment settings. The key findings indicate that patients frequently reported perceived negative attitudes from clinicians, such as judgment or dismissiveness, which adversely affected their treatment experiences. This perceived stigma resulted in reduced engagement in treatment, weaker therapeutic relationships, increased barriers to accessing care, and reinforcement of harmful weight-related beliefs. Patient narratives in these studies illustrate how perceived clinician stigma can contribute to, and perpetuate, feelings of shame, frustration, and isolation. Although research on this topic is limited, the studies reviewed help us start to understand the impact of perceived clinician stigma on patient experiences. In addition to reducing stigma, future research should focus on how clinician behaviours can positively shape eating disorder treatment experiences. Specifically, healthcare providers should focus on making patients feel heard, respected, and understood to improve treatment outcomes and foster a more supportive environment for recovery.

## Introduction

Eating disorders (EDs) are complex mental health conditions that necessitate a multidisciplinary approach to ensure effective treatment [1]. This approach, integrating expertise from psychiatry, psychology, dietetics, and medicine, aims to provide comprehensive, person-centred care tailored to the intricate needs of patients [2–5]. Despite significant advancements in mental health care, gaps remain in understanding and delivering ED treatment, particularly within primary care settings, which suggests the need for continued enhancement of treatment strategies to better serve individuals with EDs [6]. These gaps are often exacerbated by public perceptions and media representations of EDs, which tend to reinforce stereotypes and misconceptions [7–9]. EDs are frequently depicted as affecting primarily young, thin, Caucasian females, neglecting the broader demographics of those impacted. Such portrayals also commonly frame EDs as personal failings rather than recognising them as conditions requiring specialised medical and mental health support. This contributes to stigma, leading to discrimination, blame, and shame for individuals with EDs [8, 10, 11].

Misconceptions about EDs can perpetuate harmful stereotypes and may hinder effective treatment. For example, large-scale surveys in the UK have shown that public opinions towards people with EDs are consistently negative, with around a quarter of respondents believing EDs are self-inflicted; compared to only 6% holding similar views regarding schizophrenia [12]. Media representations often focus on extreme thinness as a criterion for severity, minimising the experiences of those who do not fit this narrow image [13, 14]. This skewed portrayal reinforces unhealthy attitudes towards food, weight, and body image, further stigmatising individuals with EDs [9, 15]. Clinicians are not immune to these societal views and can also develop biases which shape their perceptions and treatment approaches.

Clinician biases may lead to limited views of what constitutes an ED, and also exclude individuals who do not fit stereotypical images of someone with an ED, such as people living in larger bodies [4, 13, 16–18]. Research indicates a significant delay, averaging four years, between the onset of disordered eating symptoms and first treatment, with some cases extending to ten or more years [19]. This delay often results in adults presenting with longstanding EDs that were undiagnosed or untreated during adolescence or early adulthood. The peak age of onset for an ED is during adolescence and emerging adulthood (up to approximately age 25), with the average duration of untreated EDs ranging from 2.5 years for anorexia nervosa to 6 years for binge eating disorder [20]. Individuals with binge eating disorder or bulimia nervosa, in particular, often encounter weight stigma from general practitioners or primary care physicians, who may focus on weight loss advice rather than appropriate ED treatment [21, 22]. Such weight-focused approaches can lead patients with EDs to receive treatment primarily for weight concerns, instead of more suitable interventions which are specific to ED-related issues [21]. Such biases can thus contribute to inadequate treatment and reinforce internalised stigma among individuals, particularly those in “normal” or “larger” bodies. Further, these biases can also exacerbate the overall treatment experience for adults, who often face unique stigma-related barriers compared to younger individuals [19].

Additionally, individuals with restrictive EDs often encounter dismissal if their body mass index (BMI) fails to meet perceived diagnostic thresholds, perpetuating misconceptions about the severity of their condition, or worthiness of receiving treatment [23]. This narrow focus on weight gain rather than psychological and physiological factors underpinning restrictive EDs can lead to feelings of invalidation among patients [24]. Winkler et al. [25] reported discrepancies between patient-reported and clinician-reported outcomes, where clinicians frequently equated ED recovery with weight gain, while patients felt that their broader symptoms were overlooked. This focus on weight can hinder the development of a supportive therapeutic alliance, crucial for effective and sustained recovery [26]. Men seeking help for EDs can face additional challenges due to misconceptions that EDs exclusively affect girls and women. Historically, healthcare systems have been designed to cater to adolescent girls with restrictive-type disorders, contributing to stigma around help-seeking for males with EDs [27]. This can result in the minimisation or invalidation of EDs in men, while clinicians may also reflect broader societal attitudes that perceive EDs as less severe when experienced by males [22, 28–30]. The misconception that EDs only impact girls and women can further lead to dismissive attitudes and a lack of awareness about EDs in boys and men, which is aligned with studies reporting that men often expressed concerns about being overlooked and invalidated in ED care [22, 28–30]. Societal norms that emphasise traditional masculine ideals of strength may exacerbate stigma, as EDs might be viewed as a sign of weakness contrary to these ideals [31–33]. This invisibility and lack of awareness about EDs in men within both society and clinical practice can hinder timely diagnosis and appropriate treatment.

Broader literature on mental health services has found that patients often report that perceived clinician stigma has detrimental impacts, including increased dropout and relapse rates among patients [10, 11]. Given dropout rates for individuals with EDs are high, ranging from 20 to 51% in inpatient settings and from 29 to 73% in outpatient settings [34], it is possible that clinician stigma similarly impacts treatment outcomes in this population. Individuals with EDs frequently encounter dismissive attitudes from healthcare providers, such as patients being praised for weight loss, receiving oversimplified advice like “*eat more*” or “*diet*,” or having their concerns minimised because they do not fit the stereotypical profile of someone with an ED [8, 9, 13, 16, 35]. These dismissive attitudes and misunderstandings may perpetuate stigma and hinder effective treatment engagement, leading to reduced trust in healthcare providers and reluctance to continue treatment. Such experiences not only impact immediate care but also contribute to long-term challenges in managing EDs effectively.

While clinician stigma towards EDs is gaining increased research attention, to date, much of the literature has concentrated narrowly on health professionals’ attitudes toward EDs [3]. Such studies suggest stigmatising or unfavourable views towards individuals with EDs are common, characterised by perceptions of being difficult, blame-worthy, or attention-seeking, particularly among general practitioners [9, 14, 17]. However, there remains a notable gap in understanding patient perspectives and experiences regarding how stigma impacts their treatment trajectories. Whilst previous research has predominantly centred on clinician perspectives, there has been a paradigm shift in ED treatment and research, emphasising the value of the lived experience voice and positioning the patient at the forefront of their own care, rather than adhering to a traditional, clinician-dictated model [36–38]. Consequently, an in-depth understanding of patient perspectives is necessary for advancing treatment modalities and ensuring that care strategies are genuinely attuned to the needs and lived experiences of individuals with EDs.

Recent reviews provide some insights into the effects of stigma on ED treatment and recovery, while also revealing specific gaps in the literature [6, 39]. For example, Foran et al. [39] conducted a quantitative systematic review to explore the relationship between stigma and ED outcomes. They identified a need for research designs that establishes causality and generalises findings, noting that most existing studies are correlational and limited by small sample sizes. Daugelat et al. [6] performed a systematic review focusing on the psychological barriers to treatment engagement, particularly the roles of stigma, shame, and guilt. Their review found that these emotional responses are pervasive among individuals with EDs and act as significant deterrents to seeking help. They suggest the need for more nuanced research that dissects the different dimensions of stigma and how they interact with personal and societal factors. Despite these contributions, there remains limited understanding of how various forms of stigma impact treatment and recovery trajectories from the perspective of patients.

This review aims to address these gaps by synthesising the available research on perceived clinician stigma and its impact on adult ED treatment particularly. Adult patients face unique stigma-related barriers and distinct treatment challenges that require tailored approaches [19, 20]. For instance, older adults may experience stigma due to assumptions that EDs are confined to youth which can result in minimisation of their condition and inadequate treatment responses. This underscores the need for age-appropriate and stigma-sensitive treatment strategies. The objective of this review is to critically appraise and synthesise research on how perceived clinician stigma affects the treatment experiences of adult patients with EDs. It will explore various forms of stigma, including weight stigma, blame, and gender biases, and examines their effects on treatment engagement, recovery trajectories, and overall well-being.

## Methods

The systematic review was conducted adhering to the Preferred Reporting Items for Systematic Reviews and Meta-Analyses (PRISMA) guidelines, focusing on studies that explored the impact of perceived clinician stigma on patient experiences in the treatment and management of EDs (PROSPERO Registration: CRD42024512723).

### Eligibility criteria

The inclusion criteria were structured following the PICO framework (Participant, Intervention, Comparison and Outcomes) as proposed by Liberati et al. [40]. Eligible studies were quantitative, qualitative and mixed-methods studies that focused on adults aged 18 years and older, diagnosed with any type of ED, receiving treatment or management from healthcare professionals in any setting. Eligible studies were also required to address clinician stigma experienced in various healthcare contexts, such as outpatient, inpatient, specialised ED care, and community-based settings. Clinician stigma in this context could reference concepts of discrimination, prejudice, weight stigma, derogatory language, and dismissive attitudes. Studies were also required to address patient experiences related to ED treatment, such as satisfaction with received treatment, perceived quality of care, treatment adherence rates, therapeutic alliance between patients and healthcare providers, and psychological well-being outcomes. Eligible studies were published in English from the 1 January 2000 with an end date of 24 March 2024. The start year was selected to ensure the inclusion of recent and relevant research, reflecting contemporary practices and attitudes.

Exclusion criteria were participants under 18 years of age, lacking formal diagnoses (current or past) of EDs, not addressing clinician stigma, or primarily focused on patient outcomes without examining clinician attitudes or behaviour. Non-peer-reviewed articles, commentaries, editorials, letters, perspectives, protocols, animal studies, case studies, abstracts, conference abstracts, or posters were excluded. Grey literature, which included case studies, case series, conference proceedings, study protocols, speeches, videos, community plans, and review studies, were also excluded from this systematic review. This decision was made to maintain a rigorous focus on primary research that employed observational and descriptive study designs. Primary research provided direct empirical evidence on the impact of clinician stigma on patient experiences within various healthcare settings [41].

### Search strategy

Four electronic databases (MEDLINE, EMBASE, PsycINFO, and the Cochrane Central Register of Controlled Trials [CENTRAL]) were searched on 24 March 2024. These databases were chosen for their extensive coverage of psychology and health-related research. The strategy involved identifying key search terms and constructing search queries tailored to each database’s unique indexing system and search functionalities. These strategies were designed to capture studies that specifically addressed patient perspectives on clinician stigma in the treatment and management of EDs, incorporating terms related to EDs, clinician attitudes, treatment settings, and patient-reported outcomes. Appendix 1 details the specific search strings used in each database.

### Review strategy

Duplicate records were identified and removed using EndNote, Covidence, and manual inspection. After removing duplicates, the first author (GL) screened all abstracts and titles for eligibility, while the third author (AR) screened a subset (10%) of records. Subsequently, the primary author independently screened all full-text articles (GL), with the third author (AR) screening a portion (10%) of the full-text studies in accordance with previous review protocols [40, 42]. Any discrepancies between the authors were resolved through discussion [40].

### Data extraction

Data from each study were extracted in accordance with the specified headings sections outlined in Table 1. These headings included: Author (year), Design, Aims, Setting and Location, Symptom Level, ED Type, Sample Characteristics, Method of Data Collection, and Main Results. The extraction process was conducted by the first author (GL) to maintain consistency, with the second author (ANF) independently extracting data from a subset of the studies (10%) [42] to ensure reliability and validate the findings. Each study was appraised to extract key findings regarding how clinician stigma may impact patient experience in treating and managing EDs. This assessment included examining and systematically documenting all study designs and methodology, specific aims and objectives, clinical settings and geographical locations, detailed sample characteristics, including symptom levels, specific types of EDs experienced. The main results from each study were documented, outlining the key findings and impact of the studies.

**Table 1:** Characteristics of included studies

Study no.	Author (year)	Design	Aims	Setting and location	Symptom level	Eating disorder (ED)	Sample characteristics	Method of data collection	Main results
1	Bye, A., Shawe, J., Bick, D., Easter, A., Kash-Macdonald, M., & Micali, N. (2018)	Mixed Methods* (only quantitative reported as related to patient experience)	To explore the perceived barriers to disclosure and identification of ED in pregnancy and postnatally by women with past or current ED.	Women were recruited from a national parenting website (Netmums), United Kingdom	Current or past ED	AN (*n* = 34; 34%), BN (*n* = 16; 16%), BED (*n* = 24; 24%), EDNOS (*n* = 25; 25%) and missing data (*n* = 2)	**Gender**: Female (*n* = 101) **Age**: <24 (*n* = 21), 25–35 (*n* = 55), > 36 (*n* = 24), Missing data (*n* = 1) **Pregnancy Status**: Majority were not pregnant (post-natal) **History of EDs**: Had experienced an eating disorder in the past or currently (*n* = 92) **ED Symptoms During Pregnancy**: Experienced eating disorder symptoms during pregnancy (*n* = 64; 63%)	Survey	**Findings**: High rates of ED symptom non-disclosure (70%) due to stigma, shame, and fear of judgment. **Impact**: Clinician stigma leads to treatment avoidance and delays in care, as patients fear judgment and prefer self-management. This negatively affects patient satisfaction and psychological well-being, contributing to inadequate therapeutic alliances
2	Chen, C., & Gonzales, L. (2022)	Quantitative	To develop and validate a patient-centred assessment of weight stigma in the treatment environment to capture perspectives of provider behaviour and bias that providers may be unaware of	Multiple settings (in-patient, out-patient and in community) recruited through Amazon’s Mechanical Turk program (MTurk), United States	ED diagnosis (not specified past or current) and had received behavioural health services to treat an ED within the last 5 years	BED (*n* = 109; 76.8%), AAN (*n* = 17; 12%), BN (*n* = 12; 8.5%), and EDNOS (*n* = 3; 2.1%)	**Sample size**: *n* = 142 **Gender**: Males (*n* = 77, 54.2%), Females (*n* = 62, 43.7%) **Mean Age**: 34.16 years (SD = 8.81) **Ethnicity**: Caucasian/European American (*n* = 114, 80.3%) **Household Income**: $25,000 - $50,000 (*n* = 40, 28.3%), $50,000 - $75,000 (*n* = 41, 28.9%) **Education**: Bachelor’s degree (*n* = 73, 51.4%) **Recent Treatment History**: Received dietary counselling (*n* = 104; 73.2%) and individual psychotherapy (*n* = 85; 59.9%) within the last 5 years	Survey	**Findings**: High prevalence of provider stigma; recommendations for dieting and support for disordered eating behaviours; symptoms often overlooked **Impact**: Clinician stigma results in poor treatment adherence and avoidance, as patients feel their symptoms are not taken seriously. This diminishes patient satisfaction and hampers the development of a strong therapeutic alliance
3	Evans, E. J., Hay, P. J., Mond, J., Paxton, S. J., Quirk, F., Rodgers, B., Jhajj, A. K., & Sawoniewska, M. A. (2011)	Mixed Methods	To investigate the experiences of help-seeking in an epidemiologically derived sample of community women with EDs who had previously not been seeking treatment and who were prompted to seek treatment as part of a 4-year longitudinal study	Community setting with participants recruited form the Health and Well-Being of Female Australian Capital Territory Residents Study., ACT, Australia	Current symptoms	BN: *n* = 7 (12%); BED, *n* = 8 (14%); EDNOS (defined as having extreme weight and/or shape concerns alongside regular ED behaviour throughout the preceding 3 months), *n* = 30 (53%)	**Sample Size**: *n* = 57 participants **Mean Age**: 33 years **Employment**: 79% in paid employment **Marital Status**: 51% married or in a de-facto relationship **Parental Status**: 46% had children **Nationality**: 88% Australian born **Language**: 93% spoke English as their first language	Survey and Interview	**Findings**: Clinician stigma noted as a barrier; disappointment with professional responses; service rationing issues. **Impact**: Stigma from clinicians discourages open discussion of ED symptoms, leading to frustration and dissatisfaction. The lack of adequate time and resources hinders effective treatment, negatively affecting treatment adherence and the therapeutic alliance
4	Gulliksen, K. S., Espeset, E. M., Nordbo, R. H., Skarderud, F., Geller, J., & Holte, A. (2012).	Qualitative	To identify and delineate personal, attitudinal, and behavioural traits preferred by patients in their therapists, encompassing both satisfactory and dissatisfactory categories	Specialist ED in-patient and outpatient settings, Norway	Current symptoms	AN (*n* = 38)	**Gender**: Predominantly female participants (*n* = 38) **Average Age**: M = 28.3 years **Mean BMI**: M = 13.6 **Illness Duration**: Average M = 9.5 years **Treatment Duration**: Average M = 5.4 years	Interviews	**Findings**: Mixed positive and negative encounters with healthcare professionals; emphasis on acceptance, vitality, challenge, and expertise **Impact**: Positive experiences enhance patient satisfaction and therapeutic alliances, while negative experiences, often fuelled by stigma, lead to feelings of devaluation and increased self-blame. This reduces treatment adherence and psychological well-being
5	Harrop, E. N., Hutcheson, R., Harner, V., Mensinger, J. L., & Lindhorst, T. (2023).	Longitudinal Mixed methods	To fill a significant gap in the literature by exploring the lived experiences of individuals diagnosed with AAN, specifically focusing on their encounters with weight stigma within healthcare settings. By investigating these experiences comprehensively, the study sought to provide insights that can inform more compassionate and effective healthcare practices for this patient population	ED Treatment settings, United States of America	Current or previous	AAN (*n* = 38)	**Gender**: Predominantly female-born participants (*n* = 38), including cisgender women (*n* = 30) and non-binary/transgender individuals (*n* = 8) **Average Age**: M = 35.6 years	Survey and Interview	**Findings**: Persistent weight stigma from childhood through lifespan of ED treatment; providers pathologize weight and deny symptoms **Impact**: Weight stigma contributes to treatment avoidance, delayed diagnosis, and increased relapse rates. Patients experience dissatisfaction with care and face significant barriers to developing trust and effective therapeutic relationships
6	Lazare, K., Mehak, A., & Telner, D. (2021)	Qualitative	To examine the experiences of adult patients with ED in the context of primary care. Specifically, the research aimed to investigate how these patients perceive the care provided by their family practitioners and to identify major gaps in primary care services for adult patients with EDs. Through this exploration, the study sought to provide insights that could lead to improvements in the delivery of healthcare services and support for individuals with EDs within primary care settings	Community-based treatment, Canada	Current and recovered	Not reported	**Participants**: 10 female participants aged 24 to 47 years (M = 35.90, SD = 8.01) **Ethnicity**: All participants identified as Caucasian **Eating Disorder Status**: 20% described themselves as “recovered,” while 80% had active eating disorders **Education**: All participants had some form of higher education; 60% completed some university or college courses, 20% held an undergraduate degree, and 20% possessed a Master’s degree **Length of Care**: Participants had been under the care of their current family physician for 5 to 20 years (M = 12.50, SD = 4.30)	Interviews	**Findings**: Varied patient experiences in primary care; delayed diagnoses; key qualities for ED care; systemic barriers **Impact**: Lack of clinician expertise and empathetic communication leads to dissatisfaction and treatment delays. Stigma-related barriers, such as difficulty discussing EDs, hinder adherence and negatively impact the therapeutic alliance
7	Leavey, G., Vallianatou, C., Johnson-Sabine, E., Rae, S., & Gunputh, V. (2011)	Qualitative	To understand the reasoning for patient non-attendance and failure to engage with ED services	ED clinic recruited from lists of patients who failed to attend sessions or dropped out, United Kingdom	Current symptoms	Not reported	**Participants**: 13 participants; gender and age information not reported **Ethnicity**: Caucasian British (*n* = 7), Turkish Cypriot (*n* = 1), Jewish (*n* = 1), Caucasian and Asian descent (*n* = 1), Black European (*n* = 1), Caucasian Irish (*n* = 1), and Black Caribbean (*n* = 1) **Attendance**: 11 participants never attended sessions 2 attended once and then dropped out	Interviews.	**Findings**: Non-attendance linked to psychosocial and service-related issues; negative previous experiences with healthcare services **Impact**: Stigma and negative past experiences deter treatment engagement and adherence. This creates a cycle of dissatisfaction and mistrust in healthcare providers, undermining the therapeutic alliance and psychological well-being
8	Neyland, M., & Bardone-Cone, A. M. (2019)	Mixed methods	To investigate treatment experiences among Latinx with a history of BED and/or BN, focusing on disparities in healthcare. Specifically, the study sought to gather quantitative data on the utilisation and perceived helpfulness of different treatment modalities. Additionally, the study aimed to identify barriers to treatment, with a particular focus on acculturation and factors related to mental health treatment stigma that might influence treatment experiences among Latinx	Community settings, United States of America	Current (83.7%) or past history (16.3%) of ED	BED or BN (figure not reported)	**Study participants**: 43 females identified as Hispanic/Latinx, majority Caucasian (72%), mean age 20.58 years (SD = 2.12), mean BMI 24.44 kg/m² (SD = 5.73) **Treatment experiences**: 35% received no treatment; of those treated (65%), average four different treatments sought (SD = 2.83). Most common professionals: psychologists/therapists (68%), frequent intensive treatment: intensive outpatient programs (25%)	Survey and interview	**Findings**: Barriers to treatment utilisation include financial burden, societal stigma, and mental health stigma **Impact**: Societal and mental health stigma deter treatment-seeking behaviours and reduce patient satisfaction. Financial constraints exacerbate these issues, leading to poor adherence and weaker therapeutic relationships
9	Rance, N., Moller, N. P., & Clarke, V. (2017)	Qualitative	To understand the treatment experiences of women with a formal or self-diagnosed history of AN	Community, in-patient, out-patient, charity counselling and private practice, United Kingdom	Current, semi-recovered and recovered	AN Formally diagnosed (*n* = 11); long-standing restriction with no formal diagnosis by NHS (*n* = 1)	**Gender**: All participants were female (*n* = 12) **Age**: Participants had a mean age of 31.5 years **Disorder duration**: Average of 13.3 years	Interview	**Findings**: Dissatisfaction with treatment focus on food and weight; structural issues in AN treatment **Impact**: Clinician stigma, seen in a narrow focus on symptoms, leads to feelings of being misunderstood and disconnected. This dissatisfaction affects treatment adherence and the therapeutic alliance, reducing overall psychological well-being
10	Reyes-Rodriguez, M. L., Ramirez, J., Davis, K., Patrice, K., & Bulik, C. M. (2013)	Qualitative	To explore the facilitators and barriers that contribute to or prevent engagement or retention of Latinx in ED treatment	Community mental health, United States of America	Previous and current	BN (*n* = 3); BED (*n* = 1), binge-eating behaviour (*n* = 1)	**Gender**: All participants identified as female (*n* = 5) **Age**: Participants’ ages ranged from 26 to 38 years (M = 31.2 years, SD = 4.6) **Ethnicity**: All participants identified as Latinx (*n* = 5) **Immigration status**: Four participants were immigrants from various countries in Latin America, residing in the U.S. for periods ranging from 3 to 33 years. Two participants were undocumented immigrants, and one was born in the U.S. to first-generation immigrant parents **Treatment status**: Two participants were actively enrolled in treatment for their eating disorders, two had been referred for treatment, and one participant self-reported as recovered	Interview	**Findings**: Challenges related to clinician stigma for Latinx patients; lack of information and bilingual treatment **Impact**: Stigma and lack of cultural competence in treatment exacerbate feelings of exclusion and misunderstanding, leading to poor patient satisfaction and reduced treatment adherence. This highlights the need for culturally sensitive care to strengthen therapeutic alliances
11	Salvia, M. G., Ritholz, M. D., Craigen, K. L. E., & Quatromoni, P. A. (2023)	Qualitative	To explore patient experiences and healthcare experiences in a sample of women with BED and Type 2 Diabetes	Specialised intensive outpatient service for BED, United States of America	Unreported, but had all previously attended a specialised treatment program for BED	BED (*n* = 21)	**Age**: Mean age of participants was 49 years (SD = 14.8). Age groups included: 18–39 (*n* = 5), 40–59 (*n* = 11), and 60 or older (*n* = 5) **Race**: Majority identified as White (*n* = 19), with Black or African American (*n* = 1) and multiple race identities (*n* = 1) **Binge Eating Frequency**: Less than once a month (*n* = 5), 1–2 times per month (*n* = 2), once per week (*n* = 4), 2–3 times per week (*n* = 3), 4–6 times per week (*n* = 2), once daily (*n* = 3), and more than once daily (*n* = 2)	Interview	**Findings**: Focus on weight in medical encounters; impact of weight stigma on care quality **Impact**: Weight stigma leads to healthcare avoidance and internalised self-blame, reducing patient satisfaction and adherence. This stigma also strains patient-provider relationships, undermining the therapeutic alliance and contributing to negative psychological effects

### Quality assessment

The Critical Appraisal Skills Programme Qualitative Studies Checklist [43] was used to critically evaluate the quality of the studies included in the review. This tool was selected to rigorously examine the quality, validity, and relevance of each study, ensuring a robust assessment of their strengths and weaknesses. The CASP Qualitative Studies Checklist was deemed appropriate because the majority of the included studies were either qualitative (*n* = 6) or mixed methods (*n* = 3). For the two quantitative studies (*n* = 2), a narrative synthesis was conducted, and thus, the CASP Qualitative Checklist was applied to maintain consistency in the appraisal process. All studies were assessed by the first author (GL), and a portion (10%) [42] of the studies were independently assessed by the third author (AR) to ensure reliability and consistency in the quality appraisal process. Details can be found in Table 2.

**Table 2:** CASP Checklist

Study	Items									
	1. Was there a clear statement of the aims of the research?	2. Is the methodology appropriate?	3. Was the research design appropriate to address the aims of the research?	4. Was the recruitment strategy appropriate to the aims of the research?	5. Was the data collected in a way that addressed the research issue?	6. Has the relationship between researcher and participants been adequately considered?	7. Have ethical issues been taken into consideration?	8. Was the data analysis sufficiently rigorous?	9. Is there a clear statement of findings?	10. Is the research findings contributing to the body of literature?
1	Yes	Yes	Yes	Yes	Yes	Can’t Tell	Can’t Tell	Yes	Yes	Yes
2	Yes	Yes	Yes	Yes	Yes	Can’t Tell	Can’t Tell	Yes	Yes	Yes
3	Yes	Yes	Yes	Yes	Yes	Can’t Tell	Can’t Tell	Yes	Yes	Yes
4	Yes	Yes	Yes	Yes	Yes	Yes	Yes	Yes	Yes	Yes
5	Yes	Yes	Yes	Yes	Yes	Yes	Yes	Yes	Yes	Yes
6	Yes	Yes	Yes	Yes	Yes	Yes	Yes	Yes	Yes	Yes
7	No	Yes	Yes	Yes	Yes	Can’t Tell	Can’t Tell	Can’t Tell	Yes	Yes
8	Yes	Yes	Yes	Yes	Yes	Can’t Tell	Can’t Tell	Yes	Yes	Yes
9	Yes	Yes	Yes	Yes	Yes	Can’t Tell	Yes	Yes	Yes	Yes
10	Yes	Yes	Yes	Can’t Tell	Yes	Can’t Tell	Yes	Yes	Yes	Yes
11	Yes	Yes	Yes	Yes	Yes	Can’t Tell	Yes	Yes	Yes	Yes

### Synthesis and analysis

The data were synthesised using both qualitative and quantitative approaches. The qualitative analysis combined thematic and narrative analysis, while quantitative findings were summarised descriptively in a narrative format where formal meta-analysis was not feasible, allowing for the identification of patterns and trends across studies. Thematic analysis was conducted inductively, allowing recurring themes related to patient experiences of clinician stigma to emerge organically from the data. The first author (GL) led the coding process, examining quotes, theme titles, and broader findings from the selected studies, with themes refined through an iterative process to ensure consistency. To ensure reliability and validate the findings, second author ANF independently extracted data and conducted coding on a subset (10%) of the studies. Narrative analysis complemented this by focusing on the direct quotes and personal stories of participants, exploring how they constructed their experiences of stigma and interactions with clinicians within broader societal and personal contexts. This approach provided a deeper understanding of the lived experience of stigma, highlighting how it was perceived and articulated in treatment experiences. Both analyses were conducted from a social constructionist theoretical standpoint [44, 45], emphasising the influence of social and cultural norms on clinician attitudes and behaviours.

Quantitative data were summarised using narrative synthesis, which is a valuable method for synthesising quantitative data where meta-analysis is not appropriate [46, 47]. The focus was on the relationship between clinician stigma and outcomes such as patient satisfaction, therapeutic alliance, and treatment adherence. Key statistical findings, such as correlation coefficients, were examined to demonstrate the impact of clinician stigma on treatment engagement and recovery. However, the quality of the quantitative data from several studies was limited. In many cases, mixed-methods designs included survey data that lacked accompanying supplementary materials or detailed reporting. This resulted in missing statistical analyses or insufficient context, making it difficult to fully interpret the numerical findings related to stigma and barriers to care.

#### Reflexivity and researcher positionality

A reflexivity statement was incorporated into the analysis to account for researcher positionality and its potential influence on the findings. The first author (GL) led the data extraction and analysis for this study. GL is an eating disorders counsellor and researcher with lived experience of ED care and identifies as Australian-Polish cisgender woman in her late 30’s. While GL’s background brings valuable insights into the research process, she acknowledges that her personal experiences may introduce potential bias, particularly in the identification of themes related to stigma. GL recognises that the conclusions drawn from the data represent one possible interpretation, shaped by her own lived experience and perspective as both a researcher and someone with direct experience in the ED field.

Third author AR reviewed a subset of abstracts, full-text articles, and conducted part of the CASP quality assessment. AR is an Australian, South-Asian cis woman in her early 20s and works as a research officer at a university. AR does not have lived experience of eating disorders or body image concerns. She has significant experience in qualitative data analysis. Her position as an external observer provides objectivity; however, it also poses challenges in fully appreciating the intricacies of lived experiences. AR was mindful of this limitation, approaching the data with a critical awareness of potential biases and ensuring the integrity of the findings through reflective engagement.

Second author ANF undertook data extraction and synthesis for a subset of studies. She is an Australian, South-Asian cis woman in her late 20s, currently studying a Graduate Diploma of Psychology and working as a research officer at a university. ANF does not have lived experience of eating disorders, but her prior research in the field of eating disorders and body image provided her with a comprehensive foundation for engaging with the data. She recognised that the conclusions drawn from the data represent one possible interpretation, shaped by her training and experience. ANF was careful to remain reflexive throughout the data synthesis process, acknowledging her external position and ensuring that the lived experiences of participants were represented with rigour and sensitivity.

## Results

**Fig. 1:**
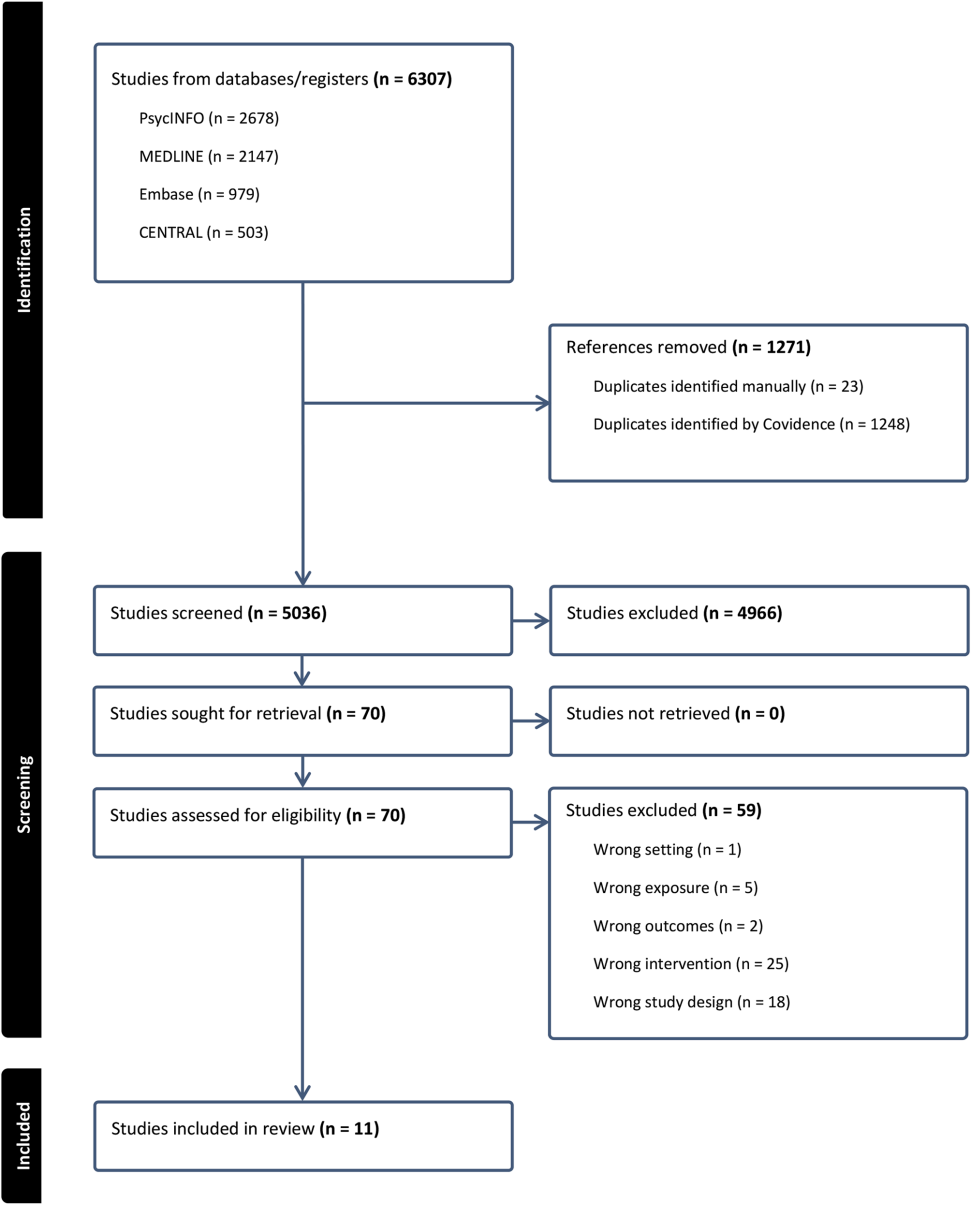
PRISMA diagram

The search terms yielded a total of 6,307 records across all databases. Following removal of 1,271 duplicates, 5,036 records remained for title and abstract screening. During this screening phase, 4,966 studies were excluded, resulting in 70 studies advancing to full-text review. After evaluation of the full texts, 11 studies met the criteria for inclusion in the review. Reasons for exclusion are outlined in Fig. 1.

### Quality assessment

Results of the quality assessment of includes studies are presented in Table 2. The overall quality of the studies was moderate. The majority of studies (*n* = 8) did not meet all the methodological quality criteria, primarily due to factors such as the inability to determine whether the relationship between researchers and participants had been adequately considered, as well as insufficient detail regarding ethical considerations. These issues contributed to moderate quality ratings, particularly in areas where reflexivity was lacking or where small sample sizes limited the robustness of the findings. Despite these limitations, no articles received a “No” in any of the CASP quality criteria. A limited number of studies (*n* = 3) completely satisfied all the quality criteria, demonstrating high or generally robust methodological standards.

### Study characteristics

Key characteristics of the 11 included studies are detailed in Table 1. The studies were conducted across five countries, with the most conducted in the United States (*n* = 5). The primary study design was qualitative (*n* = 6), followed by mixed methods (*n* = 4) and quantitative (*n* = 1). Across the studies, including those employing mixed-methods, data collection involved interviews (*n* = 9) and surveys (*n* = 5). Perceived clinician stigma toward EDs was defined and measured through various approaches across the studies, including self-report surveys and qualitative analyses of patient experiences. All studies (*n* = 11) involved participants with a current or previous ED diagnosis. Participant ED diagnoses were predominantly mixed across samples (*n* = 5), followed by participants with anorexia nervosa exclusively (*n* = 2). Sample sizes varied widely, ranging from 5 to 142 participants, with the total sample of all studies being 480 participants. Sample size was influenced by study design with smaller samples typically observed in qualitative studies. The majority of participants were cisgender women, with most studies reporting a representation of over 70% women. Participant ages ranged from 18 to 55 years (*M* = 28.3, *SD* = 8.01). Ethnicity and race were reported in 10 studies, with Caucasian participants comprising the majority in nine studies (> 80%). The studies encompassed diverse settings and countries: US multiple in-patient, out-patient and community settings (*n* = 6), Australia and Canada community settings (*n* = 2), Norway and UK specialist ED settings (*n* = 2), and UK maternity care (*n* = 1),

### Synthesis of findings

#### Qualitative studies

Qualitative studies (*n* = 6), with the addition of qualitative elements of mixed-methods studies (*n* = 3), identified three key themes illustrating impacts of clinician stigma on different dimensions of ED treatment: treatment engagement, therapeutic alliances, and barriers to treatment.

##### Treatment engagement

Across most studies (*n* = 9), clinician stigma was reported to have impacted on patient treatment engagement. Patients reported that perceived clinician stigma discouraged open discussions about their ED symptoms, led to frustration, dissatisfaction, increased ED symptomatology, and reduced treatment adherence [23, 48–55]. For example, a woman participant in one study described her therapist as evaluating her statements based on whether they aligned with the clinician’s perception of a “typical anorexic profile” [23]. When their experiences did not conform to these expectations, the clinician dismissed her concerns (“*No*,* because anorexics don’t do that*.“) (23 p589), which suggested rigid adherence to stereotypical profiles leading to a likely invalidation of patient experiences and lack of effective support.

Clinician weight-related stigma as perceived by patients was also described as contributing to treatment avoidance, delayed diagnoses, and increased relapse rates [50, 55]. A woman participant in another study reported that when she expressed concerns about having an ED, her doctor suggested she could potentially lose a little more weight [50]. She also described “…*that I was sick… but I just wasn’t sick enough. I wasn’t physically emaciated or thin enough to be considered*” (p. 55), which illustrated how the clinician’s focus on physical appearance, rather than the patient experience, likely undermined treatment and contributed to feelings of inadequacy and invalidation.

##### Therapeutic alliances

Perceived stigmatising interactions by clinicians, notably general practitioners, psychiatrists, and in-patient staff, were reported by patients to influence therapeutic alliance and patient satisfaction (*n* = 7). Results from the studies reported negative patient experiences in their interactions with clinicians, with varying attributions to stigma. In some studies, participants directly identified clinician behaviours as stigmatising, which led to feelings of invalidation and weakened therapeutic relationships; whereas, in other studies, stigma was inferred by the authors based on the context of clinician interactions [23, 49–52, 54, 55]. This distinction, between direct participant attribution and author inference, affects how the impact of clinician interactions is interpreted and understood. Harrop et al. [50] identified that participants who disclosed EDs and requested their providers avoid discussing weight loss often faced adverse responses, including repeated recommendations to lose weight and being labelled as “*non-compliant*.” For example, a participant in one study explained that “*Doctors quite literally do harm by prescribing a diet even when there is a [expletive] ED listed in my medical chart*.” (50 p57).

Similarly, stigma also played a significant role in women’s reluctance to disclose their ED to healthcare professionals, particularly during pregnancy [56]. Many women expressed feelings of shame, embarrassment, and fear of judgment. One participant highlighted how her BMI classified her as overweight, and she felt that her healthcare providers would not believe she had an actual problem: “*I was patronised by more than one healthcare professional who tried to educate me on nutrition. I got the impression they thought I was just lazy and ate junk food all of the time when this wasn’t the case. I felt they were too judgmental to approach*” (56 p.5). Further, women expressed concerns that disclosing their ED might result in unwanted referrals to child welfare services: “*I would have been too worried to discuss with my midwife for fear of being reprimanded for it”* (56, p5). Some participants also described a lack of opportunity to discuss their ED, as healthcare professionals rarely inquired about it: “*They didn’t ask*,* and it wasn’t raised as a concern*” (56, p5). Consistent with general and pregnancy-related ED research [57, 58], women often reported feeling reluctant to disclose their EDs to healthcare providers due to the perceived stigma and fear of negative consequences [56].

##### Barriers to treatment

Several studies (*n* = 5) identified specific barriers to treatment exacerbated by perceived clinician stigma. For example, both Neyland et al. [53] and Reyes-Rodriguez et al. [54] identified clinician stigma, cultural discrimination, societal stigma, and the limited availability of bilingual (particularly Spanish) treatment services as significant barriers to effective care for patients in the United States. Further, Neyland et al. [53] also noted that Latinx were less likely to be referred on for treatment when compared to their Caucasian counterparts. For example, a Latinx patient in the U.S. recounted her experience of disclosing struggles with bingeing and purging to a general practitioner, expressing her belief that she was experiencing bulimia nervosa [53]. However, due to the patient’s limited English proficiency, the general practitioner did not fully understand the severity of the issue or provide appropriate intervention. Instead, no advice or support was given, overlooking the underlying ED symptoms and psychological distress the patient was experiencing.

Stigma and shame emerged as significant barriers to treatment, with participants frequently reporting that these factors hindered their engagement with care [48, 51–53]. For example, one participant shared, “*He thought I was a weak person and couldn’t see beyond the ED* (48 p277),” illustrating how personal judgments from clinicians could exacerbate feelings of shame and hinder treatment access. Additionally, participants expressed a fear of judgement and perceived stigma, which prevented them from disclosing their issues. This fear was often compounded by a sense of disappointment or frustration when their EDs were not taken seriously or were dismissed by healthcare professionals [48]. Additionally, Lazare et al. [51] reported that more than half of their 10 participants faced significant barriers to care due to a narrow focus by healthcare providers on physical symptoms, such as weight or heart rate, often at the expense of addressing psychological aspects of EDs, such as trauma, lack of coping skills or neurodiversity. Participants described receiving minimal support beyond occasional physical examinations and routine blood testing, with one noting, “*The ED care and support was just coming in for a physical now and then*,* and just getting some blood work done*” (51 p9).

#### Mixed methods and quantitative studies

The quantitative elements across studies (*n* = 5), including mixed methods (*n* = 4) and a solely quantitative study (*n* = 1), employed varied methodologies, including collecting demographic and survey data. However, due to the lack of consistent measurement tools and methodologies, direct comparison and comprehensive analysis of findings were not feasible. Despite these limitations, several studies reported minor findings related to weight stigma and barriers to care. Specifically, weight stigma emerged as a notable concern within the quantitative data, highlighting its impact on treatment access and patient experiences.

##### Weight stigma

Chen and Gonzales [59] developed and validated the Scale for Treatment-based Experiences of Weight Stigma (STEWS), which provides a quantitative assessment of weight-stigmatising experiences in treatment settings. Their study (*N* = 142) demonstrated a positive moderate correlation between experiences of clinician stigma and elevated ED symptomatology (*r* = 0.42, *p* < 0.001). Patients with higher stigma scores (*M* = 56.3, *SD* = 12.1) also showed increased levels of internalised weight bias (*r* = 0.31, *p* = 0.012) and decreased treatment engagement. From survey data, another study reported that 88% of 101 participants attempted weight loss independently due to weight stigma perpetuated by clinician attitudes [48].

##### Barriers to care

Several studies addressed specific issues related to stigma and treatment barriers. Neyland et al. [53] found that 35% of Latinx participants did not receive ED treatment due to perceived clinician and cultural stigma, as well as financial limitations, which were linked to lower treatment utilisation and satisfaction. Bye et al. [56] reported that 64% of pregnant women with ED symptoms did not disclose their condition to antenatal care providers due to fear of judgement and stigma. This non-disclosure can lead to inadequate care and increased risks for both maternal and foetal health.

## Discussion

This systematic review provides a synthesis of the evidence regarding the experiences of perceived clinician stigma and impacts on the treatment and management of EDs, from a patient perspective. In doing so, we provide an overview of novel preliminary insights into how stigma manifests and affects patients across clinical settings, including maternity care, community-based treatment, specialist ED clinics, and general healthcare environments. We identified a total of 11 relevant studies, most employing qualitative or mixed methods designs. These qualitative research methodologies provide patient voices, while providing exploration of their personal experiences navigating perceived clinician stigma within ED treatment. By focusing on these narratives, our review demonstrates the challenges individuals face on their individual help-seeking journey.

Unlike existing reviews, such as Ali et al. [8] which primarily address the general effects of stigma, such as the broad impact on patients’ willingness to seek help or the overall negative perception of EDs, our study focuses specifically on patient narratives and the nuanced ways in which stigma impacts their help-seeking and treatment experiences. By focusing on these detailed patient perspectives, our review provides a rich understanding of how stigma manifests in different clinical settings and how it affects patients on a deeper level. Building on the foundational work of Daugelat et al. [6] and Foran et al. [39] which examined stigma from broader and more quantitative perspectives, our study integrates qualitative insights with existing quantitative data. This approach enhances the depth of understanding regarding stigma’s impact by combining narrative accounts with empirical evidence. Our review thus not only complements but also extends previous research, offering a comprehensive and patient-centric perspective on the complexities of stigma in ED treatment and recovery.

The studies identified in this review provided preliminary evidence of how perceptions of clinician stigma can negatively impact on ED patients’ experiences of treatment, engagement and the therapeutic alliance, as well as being a barrier to treatment. According to participants, clinician stigma resulted in treatment avoidance and delays in care, as patients feared clinician judgment and preferred self-management [48, 56, 59]. The qualitative research reviewed also suggested that perceived clinician stigma had negative impacts on the therapeutic alliance, contributing to feelings of devaluation and a decline in trust toward healthcare providers [23, 49, 52]. Conversely, those same patients emphasised the importance of feeling validated, being understood and accepted, in their interactions with healthcare providers. Geller et al. [26] purports that a robust therapeutic alliance hinges on mutual respect, understanding, and collaboration between patients and providers. When this alliance is compromised by perceived clinician stigma, patients may feel their struggles are invalidated, potentially undermining their willingness to engage in treatment efforts. This can contribute to worsening mental health, including increased anxiety and depression, which are common comorbidities in individuals with EDs [48, 49, 55].

The issue of weight stigma was salient across all studies, with impacts on the treatment of individuals with EDs also commonly reported by patients. Harrop et al. [50] highlighted that weight stigma often originated in childhood and persisted throughout the adult ED treatment journey, influencing self-perception and contributing to long-term psychological harm. Within healthcare settings, weight stigma has frequently led to the minimisation of ED symptoms by clinicians, or attributions solely to weight, while disregarding underlying complexities [13]. This reductionist approach delayed care, exacerbating symptoms and compromising health outcomes. Salvia et al. [55] further noted that focusing predominantly on weight and weight loss during medical interactions neglected the multifaceted aspects of ED. Such practices not only frustrated patients but also strained provider-patient therapeutic alliance by seemingly failing to address holistic needs [50, 55]. Moreover, the emphasis on weight likely promoted harmful behaviours, with patients resorting to extreme dieting or other detrimental practices to meet perceived expectations by healthcare providers [13, 50]. Further, the cumulative impact of weight stigma and societal pressures created significant barriers to seeking and sustaining treatment, as individuals often avoided or prematurely disengaged from care due to perceived stigma or fear of judgement. This promoted a cycle of deteriorating health and increased difficulty in achieving recovery, further impacting treatment engagement and outcomes. Addressing these issues is crucial for healthcare providers to foster inclusive and supportive environments that prioritise patient-centred care and move beyond weight-centric approaches.

Patient narratives in the two studies focusing on the experiences of Latinx in ED treatment demonstrated the significant impact of cultural and language barriers in healthcare settings [53, 54]. This emphasises how cultural and language barriers can contribute to misunderstandings and inadequate responses in healthcare interactions in general. Patients from cultural minority backgrounds often face challenges in effectively communicating their health concerns, leading to underdiagnosis or misdiagnosis of EDs and other conditions [53]. Such experiences not only hinder treatment effectiveness but also exacerbate feelings of perceived stigma, alienation and distrust toward healthcare providers.

We also found negative patient experiences related to perceived clinician stigma across diverse treatment settings. While studies conducted in maternity care, community-based treatment facilities, specialist ED clinics, and general healthcare settings all documented instances of clinician stigma, the extent and focus of these studies varied [48–50, 56, 59]. Therefore, while the presence of perceived clinician stigma was documented across different contexts, the depth and specificity of the reported experiences differed, reflecting the need for more extensive research in certain areas. For instance, some studies provided detailed accounts of how specific stigmatising comments from clinicians affected patients’ willingness to engage in treatment, while others only noted general feelings of judgement without exploring the nuances of how these interactions influenced care-seeking behaviours. Despite these variations, these findings indicate that perceived clinician stigma may not be limited to one particular healthcare setting and suggest the potential need for system-level interventions to improve clinician awareness, sensitivity, and overall quality of care for individuals with EDs worldwide.

### Clinical implications

Interventions aimed at reducing stigma among healthcare workers have primarily focused on education and social contact strategies, with varying degrees of success in changing attitudes and behaviours [60–63]. Healthcare settings remain significant sources of stigma for individuals with mental illness globally [61]. Of the limited interventions examined for healthcare workers, those incorporating multiple forms of contact, such as live or filmed mock interactions, show more favourable outcomes in mental health knowledge and attitudes compared to educational interventions alone. Specifically, interventions that included personal testimonies and multiple social contact elements were effective in improving healthcare workers’ empathy, understanding of mental health conditions, and reduction in stigmatising attitudes. Standardised role-plays have also shown promise in reducing stigma among healthcare students and professionals by simulating real patient interactions, helping practitioners practice respectful communication and empathy, improving their understanding of patient experiences and fostering more sensitive responses [60, 61]. Given the mixed efficacy of stigma reduction interventions across healthcare workers, more research is crucial to identify the most effective strategies for sustained stigma reduction and improved patient care, particularly in ED settings.

Beyond the reduction of stigma, it is imperative to focus on the dimensions of clinician care that actively facilitate positive treatment experiences, particularly from the perspectives of individuals with lived experience. Core to this is the importance of patients feeling heard, respected, and understood; factors which are inextricably linked to enhanced treatment outcomes [8, 26]. Many clinicians may be unaware of the ways in which their practices are perceived as stigmatising, as these behaviours often emerge from implicit assumptions about health and body image. For instance, while recommending weight loss in the context of eating disorder care may be framed as a health-promoting intervention, such advice can be experienced as stigmatising by patients, exacerbating feelings of inadequacy and alienation [13, 64, 65]. Clinicians themselves are not immune to the pervasive societal discourses surrounding weight and health, which can unconsciously inform their clinical interactions and treatment strategies [17, 31, 66]. As such, it is essential that clinician training programs incorporate reflective practices that encourage healthcare professionals to critically examine and deconstruct these underlying assumptions [2]. This approach can foster a more patient-centred model of care, grounded in empathy and sensitivity to individual experiences of stigma. By cultivating an increased awareness of the subtle ways stigma may manifest, even unintentionally, clinicians can enhance the therapeutic alliance and contribute to improved treatment outcomes.

### Strengths, limitations and future research

One of the key strengths of this systematic review lies in the depth and detail of the qualitative data presented across the included studies. Many of the studies provide valuable insights into patient experiences, particularly through the integration of lived experience voices. This approach allows for a more comprehensive understanding of how clinician stigma is perceived and internalised, highlighting not only the emotional and psychological impacts but also the broader consequences for treatment engagement and recovery. Several studies also utilised diverse methodological approaches, such as interviews and focus groups, which strengthened the findings by providing multiple perspectives and triangulating data to enhance the credibility and robustness of the conclusions drawn. However, a limitation of this systematic review is its inclusion of only 11 studies, reflecting a limited pool of research on perceived clinician stigma and its impact on the patient experience of ED treatment. This restricted scope may affect the comprehensiveness of the conclusions drawn. Additionally, the limited number of studies highlights gaps in the literature, particularly regarding specific demographic groups treatment settings (e.g., older adults, LGBTQ + individuals) and treatment settings (e.g., inpatient versus outpatient care) and geographic regions (e.g., non-Western countries) that may be underrepresented. Future research efforts should aim to expand upon these findings with larger and more diverse samples to enhance the generalisability and robustness of conclusions regarding perceived clinician stigma in the context of the patient experience in ED treatment. Furthermore, this review’s findings are impacted by methodological limitations. Whilst most studies employed qualitative or mixed-methods designs, which provided valuable insights into patient experiences, they failed to include robust quantitative measures, limiting the generalisability of their findings [53–55]. To enhance comparability across studies and strengthen the evidence base, future research should employ consistent definitions and quantitative measures of stigma. The lack of clarity about how stigma was conceptualised and operationalised in different studies was a notable limitation that affects the interpretation and comparability of findings.

Additionally, the qualitative nature of the data introduces a hermeneutic dimension, where interpretation plays a crucial role in understanding lived experiences. This interpretative process, inherent in qualitative syntheses, adds subjectivity on the part of both participants and researchers. To enhance the transparency of these interpretations and provide clearer contextualisation of the results, future studies should incorporate reflexive accounts. As identified in the CASP quality assessment, greater attention to the researcher-participant dynamic is needed, as this could enhance the validity of the data and deepen the understanding of how clinician stigma is experienced. Furthermore, most studies reviewed were cross-sectional, limiting their ability to establish causality. Longitudinal studies are necessary to explore how perceived clinician stigma evolves and impacts treatment engagement, therapeutic alliance, and recovery over time. Incorporating consistent definitions and quantitative measures of stigma in future research would also strengthen comparability across studies and improve the evidence base. Addressing these methodological and conceptual gaps will provide a more comprehensive understanding of how stigma affects ED treatment experiences and outcomes.

## Conclusion

This systematic review provides novel insights into the impact of perceived clinician stigma on the treatment landscape and patient experiences for individuals with EDs. Across synthesised studies, perceived clinician stigma emerged as a substantial barrier to treatment in qualitative accounts, adversely affecting patient satisfaction, treatment adherence, and therapeutic alliances. The qualitative themes revealed how potential biases among healthcare providers may impede effective care delivery and exacerbate challenges faced by ED patients. The review also demonstrates the necessity for reliable measures of clinician stigma and patient-reported outcomes to enhance comparability across studies and strengthen the evidence base. By addressing these issues directly, healthcare systems and providers can seek to implement robust interventions aimed at mitigating stigma, enhancing provider education, and fostering environments of empathy and cultural competence. This may cultivate stronger therapeutic alliances, improve patient satisfaction, and enhance adherence to treatment protocols.

## Appendix 1. Search strategy

**Table:** 

Concept 1	AND	Concept 2	AND	Concept 3
KEYWORDS & PHRASES		KEYWORDS & PHRASES		KEYWORDS & PHRASES
Eating disorder* OR Anorex* OR Bulimi* OR Binge eating* OR Avoidant restrictive food intake OR Pica OR Rumination OR OSFED OR Atypical anorex* OR ARFID		Stigma* OR Clinician stigma* OR Clinician discriminat* OR Healthcare stigma* OR Healthcare Discriminat* OR Prejudice OR Reject* OR Ambivalen* OR Attitude* OR Bias* OR Weight bias* OR Weight stigma*		Patient experience* OR Patient satisf* OR Treatment* OR Management* OR Help* OR Barrier* OR Shame OR Recover* OR Therapeutic all* OR Psychological effect* OR Psychological impact*
SUBJECT HEADINGS		SUBJECT HEADINGS		SUBJECT HEADINGS
**MeSH (Medline)** Feeding and Eating Disorders		**MeSH (Medline)** Stereotyping OR Perceived Discrimination OR Attitude of Health Personnel OR Health Knowledge, Attitudes, Practice OR Delivery of Health Care OR Delivery of Health Care, integrated OR Weight prejudice		**MeSH (Medline)** Patient Satisfaction OR Quality of Health Care OR Help-Seeking Behavior OR Patient Acceptance of Health Care OR Shame OR Recovery of Function OR Stress, Psychological OR Treatment Adherence and Compliance OR Patient Compliance OR Therapeutic Alliance
**Emtree (Embase)** Eating disorder		**Emtree (Embase)** Stereotyping OR Perceived Discrimination OR Health Personnel Attitude OR Health Care Delivery OR Weight Stigma		**Emtree (Embase)** Patient Satisfaction OR Health Care Quality OR Help Seeking Behavior OR Patient Attitude OR Mental Health Recovery OR Mental Stress OR Psychotrauma OR Patient Compliance OR Therapeutic Alliance OR Doctor Patient Relation
**PsychInfo** Eating Disorders		**PsychInfo** Stereotyped Attitudes OR Social Discrimination OR Health Personnel Attitudes OR Therapist Attitudes OR Health Knowledge OR Mental Health Stigma		**PsychInfo** Client Satisfaction OR Client Attitudes OR Client Participation OR Quality of Care OR Treatment Compliance OR Recovery (Disorders) OR Help Seeking Behavior OR Health Care Seeking Behavior OR Shame OR Stress OR Psychological Stress OR Therapeutic Alliance OR Psychological Consequence
**CENTRAL** Feeding and Eating Disorders		**CENTRAL** Stereotyping OR Perceived Discrimination OR “Attitude of Health Personnel” OR Health Knowledge, Attitudes, Practice OR “Delivery of Health Care” OR Weight Prejudice OR Clinical Competence		**CENTRAL** Patient Satisfaction OR “Quality of Health Care” OR Help-Seeking Behavior OR “Patient Acceptance of Health Care” OR Patient Compliance OR Shame OR Mental Health Recovery OR “Recovery of Function” OR Fear OR Stress, Psychological OR Psychological Distress OR Psychological Trauma OR Therapeutic Alliance

Concepts and Search Terms for MEDLINE, EMBASE. PsycINFO, and Cochrane Central Register of Controlled Trials [CENTRAL]

* The .mp field was searched for keywords

** Range 1 January 2000-24 March 2024

## Data Availability

No datasets were generated or analysed during the current study.
